# A super-infection in the cornea caused by *Stemphylium*, *Acremonium*, and *α*-*Streptococcus*

**DOI:** 10.1186/s12941-017-0187-z

**Published:** 2017-03-09

**Authors:** Fumika Hotta, Hiroshi Eguchi, Keiko Nishimura, Masahiro Kogiso, Mayumi Ishimaru, Shunji Kusaka, Yoshikazu Shimomura, Takashi Yaguchi

**Affiliations:** 1 Department of Ophthalmology, Sakai Hospital Kindai University, 2-7-1 Harayamadai, Minami-ku, Sakai, Osaka 590-0132 Japan; 2 Department of Ophthalmology, Shikoku Medical Center for Children and Adults, 2-1-1 Senyu-cho, Zentsuji, Kagawa 765-8507 Japan; 3 Department of Ophthalmology, Kindai University Hospital, 377-2 Ohnohigashi, Osakasayama, Osaka 589-8511 Japan; 4 Medical Mycology Research Center, Chiba University, 1-8-1 Inohana, Chuo-ku, Chiba, 260-8673 Japan

**Keywords:** Keratitis, Super-infection, *Stemphylium*, *Acremonium*, *α*-*Streptococcus*, Microscopic examination, Pathogenicity

## Abstract

**Background:**

Polymicrobial keratitis with fungus and bacteria can lead to blindness and is challenging to treat. Here, we introduce a case of fungal keratitis caused by two different strains in addition to definite bacterial super-infection caused by an *α*-*Streptococcus* sp., and describe the importance of microscopic examination.

**Case presentation:**

A 74-year-old woman, who had a past history of infection with leprosy, presented with conjunctival hyperaemia, pain, and corneal opacity in her right eye. Under the presumptive diagnosis of infectious keratitis, corneal scrapings were stained by various reagents and inoculated on several agar plates. Microscopic findings of the scrapings revealed fungi and a small number of Gram-positive cocci. Multiple anti-fungal therapies with levofloxacin ophthalmic solution were administered. Although empiric treatment was initially effective, keratitis recurred 10 days after its initiation. Repeated corneal scraping revealed an abundance of Gram-positive chain cocci and a small amount of fungi, resulting in the switching of an antibiotic medication from levofloxacin to moxifloxacin and cefmenoxime. Keratitis resolved gradually after the conversion. *Stemphylium* sp., *Acremonium* sp., and *α*-*Streptococcus* sp. were simultaneously isolated from the corneal scrapings.

**Conclusions:**

To the best of our knowledge, this is the first case of fungal keratitis caused by *Stemphylium* sp., and also the first case of super-infection in the cornea caused by two different fungi and one bacterium. Microscopic examination of the corneal scrapings was beneficial in rapid decision of changing to appropriate drug according to the dominancy of pathogenicity.

## Background

Fungal eye infection is a refractory infectious disease that can lead to blindness [1]. Since the cultivation of fungi generally takes longer than that of bacteria [2], clinicians should diagnose fungal infection through cornea microscopic examination of clinical samples as soon as possible. In ophthalmology, the clinical diagnosis of fungal keratitis can easily be established when fungi are present in the corneal smear. Since mixed infections with a combination of bacteria and fungus are not uncommon [3], it is reasonable for ophthalmologists to administer topical antibiotics eye drops as an adjunctive therapy for prophylaxis of bacterial super infection, even in cases of microscopically proved fungal keratitis. The selection of antibiotics in such cases is generally not rigorous but empirical, and monotherapy with fluoroquinolone eye drops may be selected [4, 5]. The selection among quinolones may be regarded as unimportant because an antibiotic effect is not the main purpose.

Verifications of the treatment strategy and selection of antibiotics are needed if the infection does not heal as expected. In such situations, repeated microscopic examination of the clinical samples is useful for the rigorous detection of the causative microbes. However, in recent ophthalmological clinical settings, corneal scraping and Gram staining are sometimes avoided, especially among non-corneal specialists [6]. Since polymicrobial keratitis with fungus and bacteria is not uncommon and challenging to treat [7], ophthalmologists should scrape the cornea and stain it with Gram reagent.

In this case report, we describe the importance of microscopic examination, in terms of both detecting a change in pathogenicity dominance and antibiotic selection, by introducing the first known case of fungal keratitis caused by two different strains plus a definite bacterial super-infection caused by *α*-*Streptococcus* sp.

## Case presentation

**Fig. 1:**
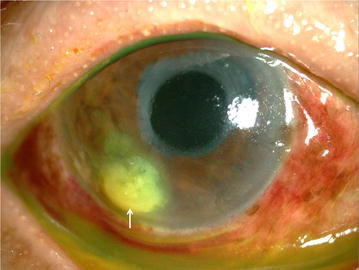
Anterior segments photographs at the initial visit. The white abscess in the temporal inferior cornea is remarkable (*white arrow*)

**Fig. 2:**
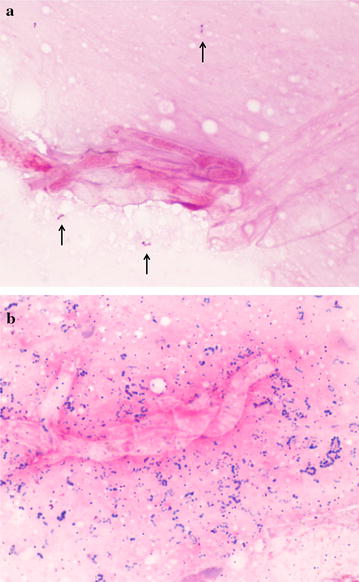
**a** Gram staining of the corneal scrapings at first visit (×1000). Fungi and a small number of Gram-positive cocci (*black arrows*) are present. **b** Gram staining of the corneal scrapings at the time of relapsing (×1000); there is an abundance of Gram-positive chain cocci present

A 74-year-old woman, who had visited the clinic regularly due to eye complications caused by leprosy, returned irregularly with eye pain and conjunctival hyperaemia in her right eye. She had lagophthalmos due to facial palsy. During her scheduled visits, she had occasionally been prescribed topical steroid for iritis. Slit lamp examination revealed infiltration and a white abscess in her temporal cornea (Fig. 1a). The corneal scrapings stained by Gram, Giemsa, and fungi flora Y reagents, showed a large abundance of fungi and very few Gram-positive cocci (Fig. 2a), led us to the diagnosis of fungal keratitis. The corneal scrapings were also inoculated on Sabouraud, potato dextrose, and sheep blood agar plates (Nissui Pharmaceutical Co., Ltd., Tokyo, Japan), and cultivated at both room temperature and at 35 °C.

Based on our presumption that Gram-positive cocci represented the commensal bacteria of the ocular surface, empirical therapy for fungal infection with 1.0% topical voriconazole eye drops (Vfend^®^, Pfizer Japan Inc., Tokyo, Japan), administered every hour and 1.0% pimaricin ointment (Pimaricin ophthalmic ointment Senju^®^, Senju Pharmaceutical Co., Ltd., Osaka, Japan), administered 4 times daily, concurrent with 100 mg/day itraconazole (Itrizole^®^ Capsules 50, Janssen Pharmaceutical K.K., Tokyo, Japan) were initiated. We also administered levofloxacin (LVFX) ophthalmic solution (Cravits^®^ ophthalmic solution 1.5%, Santen Pharmaceutical Co., Ltd., Osaka, Japan) 4 times daily for the potential occurrence of bacterial super infection. Although clinical findings improved steadily over the first 5 days, keratitis and anterior chamber inflammation recurred 10 days after therapy initiation. We repeated cornea scraping and staining using the same reagents as previously. Microscopic images revealed a large number of Gram-positive chain cocci (Fig. 2b). Therefore, levofloxacin ophthalmic solution was replaced by moxifloxacin (MFLX) (Vegamox^®^ ophthalmic solution 0.5%, Alcon Japan Ltd., Tokyo, Japan) and cefmenoxime (CMX; Bestron^®^ for ophthalmic 0.5%, Senju Pharmaceutical Co., Ltd., Osaka, Japan), whilst continuing the same antifungal medications.

**Fig. 3:**
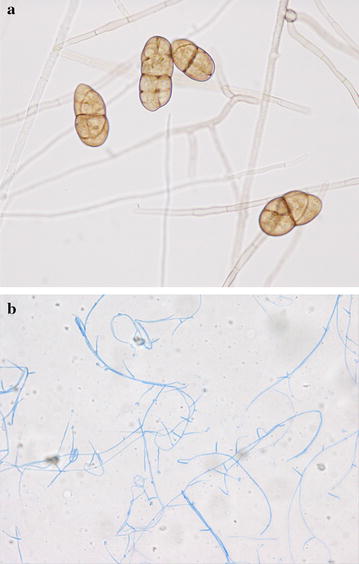
**a** Slide culture of the isolate of *Stemphylium* sp. (×400, Lactophenol staining). Conidia form a distinctive conidiophore. **b** Slide culture of the isolate of *Acremonium* sp. (×400, Lactophenol cotton blue staining). Long ellipsoidal conidia are shown

Cultivation of corneal scrapings before therapy initiation on Sabouraud and potato dextrose agar plates at room temperature resulted in the growth of two different fungi 10 days later. At the same time, the cultivation on sheep blood agar plate in 35 °C produced bacterial colonies 3 days later. One of the two isolates had solitary, darkly pigmented, terminal and multicellular conidia (dictyoconidia), formed on a distinctive conidiophore with a darker terminal swelling (Fig. 3a) and the another isolate had hyphae and long ellipsoidal conidia, aggregated in slimy heads at the apex of each phialide (Fig. 3b). The sequences of the internal transcribed spacer region of ribosomal RNA gene were analyzed by the BLAST research at the NCBI website (http://blast.ncbi.nlm.nih.gov/Blast.cgi). As a result, they showed 100% homology with sequence data of *Stemphylium* spp. strains in the former and *Acremonium* spp. strains in the later. Therefore, these two isolates were identified as *Stemphylium* spp. and *Acremonium* spp. based on their morphology and phylogeny. The minimum inhibitory concentrations (MICs) of several antifungal drugs and minimal effective concentration of micafungin for the two strains were determined by the broth dilution method according to M38-A2 of the Clinical and Laboratory Standards Institute (Table 1). A bacterium was identified as *α*-*Streptococcus* sp. and the drug sensitivity (Table 2) of the strain was determined by an automatic rapid identification machine (RAISUS, Nissui Pharmaceutical Co., Ltd., Tokyo, Japan). Keratitis resolved gradually after the conversion of topical antibiotic medications and had healed completely 3 months after the conversion (Fig. 1b).

**Table 1:** Minimum inhibitory concentrations or minimal effective concentrations of 2 fungal strains

Drugs	*Stemphylium* sp.	*Acremonium* sp.
	MIC/MEC (μg/mL)	MIC/MEC (μg/mL)
MCFG	−	0.12
AMPH	−	>16
5-FC	−	>64
FLCZ	−	>64
ITCZ	−	>8
VRCZ	−	8
MCZ	−	4

*MCFG* micafungin, *AMPH* amphotericin B, *5-FC* flucytosine, *FLCZ* fluconazole, *ITCZ* itraconazole, *VRCZ* voriconazole, *MCZ* miconazole, − not examined because the number of spores required for the measurement could not be obtained

**Table 2:** Minimum inhibitory concentrations of a strain of *α*-*Streptococcus* sp.

Drugs	*α*-*Streptococcus* sp.
	MIC (μg/mL)
PCG	0.25
ABPC	0.5
IPM	<0.125
CTX	<0.5
CTRX	<0.5
MINO	4
LVFX	>4

*PCG* benzylpenicillin, *ABPC* ampicillin, *IPM* imipenem, *CTX* cefotaxime, *CTRX* ceftriaxone, *MINO* minocycline, *LVFX* levofloxacin

## Conclusions

The current case highlights rare and important issues in the field of infectious diseases; (1) a super infection caused by two different fungi and a bacterium, and (2) microscopic examination of corneal scrapings can reveal changes in the pathogenic superiority. With respect to the former issue, rigorous cultivation of corneal scrapings and fungal species identification contributed to the current report of the first known (to the best our knowledge) case of keratitis caused by a *Stemphylium* sp. This genus comprises plant-pathogenic fungi and is widely distributed globally [8]. Although there are no reports describing its pathogenicity to humans, we presume that this strain of *Stemphylium* was transferred from an outdoor environment to the patient’s cornea because the patient lived on a solitary island in a rural area in Japan. Alternatively, this strain of *Stemphylium* may have attached to the immune-compromised ocular surface due to keratitis caused by *Acremonium* spp. Furthermore, the current case had a history of leprosy, which can have several sequelae resulting in an immunocompromised condition at the ocular surface, such as a decrease in corneal sensitivity [9] and lagophthalmos [10]. These may have affected the polymicrobial keratitis in the current case. We believe that rigorous cultivation of the corneal scrapings and species identification of isolated fungi may have contributed the discovery of this new keratitis pathogen.

In general, it is difficult for ophthalmologists to detect changes in pathogenic superiority in corneal super-infections by routine examination and clinical course reviews. In cases of intractable fungal keratitis, for which empirical therapy is ineffective, several possibilities should be considered: (1) fungal infection has persisted or recurred, (2) bacterial super-infection has occurred and the selection of the antibiotics was inadequate, (3) systemic diseases have caused an immunocompromised ocular surface that can affect the clinical course of keratitis, and (4) the patient drug adherence was poor. We ruled out (3) and (4) as causes, because no history of systemic disease that could cause an immunocompromised condition at the ocular surface was reported and we confirmed the patient’s good adherence to the eye drops. In case of (1), the selection of antifungal drugs should be reconsidered after rigorous cultivation of pathogenic fungi. In the case of (2), the selection of antibiotics should be reconsidered. However, it may be difficult to presume that only the conversion of antibiotics selection leads to keratitis healing, in cases initially determined as fungal keratitis. In such cases, microscopic examination of the scraped cornea is crucial. In the current case, we had to presume that a small number of Gram-positive cocci in the first corneal scraping represented the pathogenic strain. Despite their low numbers, microbes revealed under light microscopy should be considered as the possible pathogenic strain because they could present in human tissue to a greater extent than in the specimen.

MFLX is one of the fourth-generation fluoroquinolone agents, which have increased potency against Gram-positive bacteria compared with LVFX [11]. The MIC of MFLX for the strain of *α*-*Streptococcus* sp. was not determined in the current case because the MFLX sensitivity test plate was not prepared. However, MFLX is more effective at treating infections caused by *Streptococcus* spp. than LVFX. Gatifloxacin (GFLX), another fourth-generation fluoroquinolone agent is thought to be more effective for prophylaxis against clinical isolates of *Streptococcus pneumonia* based on a rabbit LASIK model [12]. In the current case, we considered potential bacterial invasion into the anterior chamber. Given that MFLX has a higher permeability in the anterior chamber than GFLX [13], it was reasonable to select MFLX ophthalmic solutions, as opposed to GFLX. We also selected CMX, a third-generation cephalosporin antibiotic, as an additional topical medication to MFLX. This was because it is generally sensitive to *Streptococcus* spp. according to MIC, and dual-fortified broad-spectrum antibiotics are recommended for more severe corneal ulcers [4, 5]. This is an inference because we could not test the susceptibilities of MFLX, GFLX, and CMX because the pathogenic strain was not sub-cultured and the hospital did not prepare MIC measuring plates for these drugs. However, we believe that the conversion of topical antibiotics from LVFX to MFLX with CMX, dependent on the microscopic findings, may have contributed to keratitis improvement in the current case.

In conclusion, to the best of our knowledge, this is the first case of fungal keratitis caused by a *Stemphylium* sp. It is also the first case of a super-infection in the cornea caused by two different fungi with one bacterium. This case demonstrates the significance of cornea microscopic examinations in infectious keratitis, in terms of rigorous diagnosis and detecting changes in pathogenicity dominance. This case substantiates the view that clinicians in all fields should perform microscopic examinations for intractable infectious cases.

## Abbreviations

CMX: cefmenoxime
GFLX: gatifloxacin
LVFX: levofloxacin
MIC: minimum inhibitory concentration
MFLX: moxifloxacin
